# The added value of sensor-based tests in explaining the variance in walking and ADL independency after stroke: An exploratory study

**DOI:** 10.1177/02692155251362742

**Published:** 2025-08-03

**Authors:** Natasja Charon Wouda, Marieke Geerars, Martijn Frits Pisters, Johanna Maria Augusta Visser-Meily, Michiel Punt

**Affiliations:** 1 Center of Excellence for Rehabilitation Medicine, UMC Utrecht Brain Center, University Medical Center Utrecht, Utrecht University and De Hoogstraat Rehabilitation, Utrecht, The Netherlands; 2 Department of Neurorehabilitation, De Hoogstraat Rehabilitation, Utrecht, The Netherlands; 3 Rehabilitation Center De Parkgraaf, 8110 AxionContinu, Utrecht, The Netherlands; 4 Center for Physical Therapy Research and Innovation in Primary Care, Julius Health Care Centers, Utrecht, The Netherlands; 5 Research Group Empowering Healthy Behaviour, Department of Health Innovations and Technology, Fontys University of Applied Sciences, Eindhoven, The Netherlands; 6 Department of Rehabilitation, Physical Therapy Science & Sports, UMC Utrecht Brain Center, University Medical Center Utrecht, Utrecht, The Netherlands; 7 Research group Lifestyle and Health, University of Applied Sciences Utrecht, Utrecht, The Netherlands

**Keywords:** Stroke, walking, ADL, association, IMU, sensor-based, prediction

## Abstract

**Objective:**

To evaluate the added value of sensor-based tests over conventional tests in explaining the variance in independence in activities of daily living (ADL) and independent walking in patients during inpatient stroke rehabilitation.

**Design:**

Cross-sectional design

**Setting:**

Inpatient stroke rehabilitation

**Participants:**

Data were collected from 115 patients with stroke admitted to inpatient rehabilitation.

**Intervention:**

No intervention has been applied.

**Main measures:**

Conventional measures and sensor-based tests in which postural sway and gait variables were measured using inertial measurement units. Outcome measures were ADL independence (determined by Barthel Index [BI]) and independent walking (determined by Functional Ambulation Categories [FAC]).

**Results:**

With univariable linear regression analyses showed that the Berg Balance Scale (BBS) explained most variance in ADL independence (*R*²* =* .527) and independent walking (*R*²* =* .727). In hierarchical multivariable regression analyses, symmetry during walking without a walking aid contributed most (Δ*R*²=18.6%) in explaining variance in ADL independency, resulting in a model explaining 15.7% (*p* = .029) of the variance in the BI. Tempo during walking with a walking aid contributed most (Δ*R² =* 20.1%) in explaining variance in independent walking, resulting in a model explaining 23.3% (*p* = .002) of the variance in the FAC. Adding sensor-based variables to models with the BBS did not significantly improve variance explanation.

**Conclusions:**

The added value of variables measured with an inertial measurement unit in explaining ADL independence and walking ability after stroke is limited. These findings contribute to understanding the use of inertial measurement units in stroke rehabilitation, but caution is needed when applying them to predict physical recovery.

## Introduction

Motor impairment is common after stroke and often limits independence in walking, transfers, personal care, and mobility. Beyond its impact on activities of daily living (ADL), reduced ambulation also affects participation in daily life during the chronic phase,^1^ highlighting the importance of rehabilitation to monitor and restore ambulatory function.^2^ However, recovery is difficult to measure due to stroke heterogeneity and the multitude of factors influencing recovery.^3^ A major challenge is the large individual variability in spontaneous recovery; up to 30% of patients show no spontaneous neurological recovery on the hemiplegic side.^4^ This highlights the importance of monitoring physical recovery and determining the expected recovery in patients after stroke.

Utilizing measurement instruments could enhance the prediction of long-term outcomes after stroke, aiding decision-making for patients, caregivers, and clinicians during rehabilitation.^5^ Currently, a variety of measurement instruments are employed to monitor post-stroke recovery. Conventional tests, such as the Berg Balance Scale (BBS) and Trunk Control Test (TCT), are commonly used to assess physical function at the “Activities” level of the International Classification of Functioning, Disability and Health (ICF) model.^6^ Although both show excellent inter- and intrarater reliability, they are limited by subjectivity, ceiling and floor effects, and the potential for compensation by the unaffected limb or use of aids.^7,8^ This highlights the need for more objective tools to accurately quantify physical recovery after stroke.

Recently, wearable sensors such as inertial measurement units have advanced sufficiently to enable applications in clinical practice.^9–11^ The primary advantage of inertial measurement units is their ability to objectively quantify physical functioning at the “Function” level of the ICF model,^6^ using features derived from (angular) velocity data. Felius et al. demonstrated that gait characteristics can also be reliably measured with inertial measurement units, which capture distinct gait aspects, such as speed, symmetry, and variability.^12,13^ Besides the proven clinical properties of inertial measurement units in this population, these are suitable for frequent use, not affected by ceiling or floor effects and easy applicable in clinical practice. In our opinion, the inertial measurement units can offer a complementary and objective perspective to conventional clinical assessments in prognostic modelling after stroke, especially given that current models are often not easy applicable in clinical practice.^14^ We hypothesize that inertial measurement units will add predictive value beyond conventional tests in explaining variance in ADL independence and independent walking after stroke. This study aims to assess the added value of inertial measurement unit-based measures in explaining variance in ADL independence and walking ability during subacute stroke rehabilitation.

## Methods

Data were collected by the Making Sense of Sensor Data consortium, in which the recovery of balance and gait of patients was monitored by inertial measurement units and conventional tests during inpatient stroke rehabilitation. For the current exploratory cross-sectional study, we used data collected within the first week after admission to the rehabilitation center. The study was approved by the medical ethical review committee of Utrecht, the Netherlands (METC number: 20-462/C).

Consecutive patients with stroke, admitted to five (geriatric) rehabilitation centers in the Netherlands, were eligible to participate in our study between January 2021 and July 2023. This study included all patients with first-ever or recurrent stroke, who were able to understand simple instructions and were able to understand and sign the informed consent form. The exclusion criteria were: a severe aphasia, the inability to speak Dutch or English, pre-morbid orthopedic or balance problems which could affect balance and/or gait, and the inability to sit unsupported for at least 60 s.

After checking the inclusion and exclusion criteria, patients were personally invited to participate in this study by a physiotherapist or researcher in the rehabilitation center. Patients were enrolled to the study when they signed the informed consent form. Within one week after admission to the rehabilitation center conventional and inertial measurement unit data were collected. Also, general patient characteristics, such as gender, age, stroke type, affected side, and time since stroke, were collected from the medical chart of the rehabilitation center.

### Variables

Two dependent outcome measures were used to determine the independency in ADL and walking ability in daily life. The first clinically relevant outcome measure was the Barthel Index (BI). The Dutch 10-item observation version was used to indicate the extent to which the patient is ADL independent on a scale of 0‒20.^15^ The second outcome measure was Functional Ambulation Categories (FAC) to determine the ability to walk on a scale of 0‒5.^16^ Higher scores on both the BI and FAC indicate greater independence in ADL and walking ability, respectively.

Independent variables are balance, trunk stability, and strength of the affected leg, determined by the BBS, TCT, and Motricity Index (MI), respectively. These conventional measures are performed by a physiotherapist of the respective rehabilitation center. The test set was complemented with protocolized balance and gait tests using the inertial measurement units.^9,12^ These contained two sitting, three standing and two walking tests, with increasing difficulty (see Table 1). Depending on the physical ability of the participant, the patient was able to perform one to seven tests measured with inertial measurement unit(s).

**Table 1.** Description of inertial measurement unit-based tests.

Position	Inertial measurement unit-based test	Description	Domain
Sitting	SIT	Sitting unsupported on a flat, stable surface with feet touching the ground and knees in a 90° angle for 60 s	Postural sway
	SIT UNSTABLE	Sitting unsupported on an air cushion (placed on a stable surface) with feet touching the ground and knees in a 90° angle for 60 s	
Standing	STAND	Standing unsupported with feet in self-selected position for 60 s	
	STAND EC	Standing unsupported with closed eyes and feet in self-selected position for 30 s	
	STAND FOAM	Standing unsupported on a foam balance pad (placed on the ground) with feet in self-selected position for 30 s	
Walking	2MWT_with_	Walking without physical assistance, using a walking aid for 2 min	The way the patient walks
	2MWT_without_	Walking without physical assistance and without walking aid for 2 min	

During the sitting and standing tests, the participant was equipped with one inertial measurement unit manufactured by Aemics b.v. Oldenzaal, The Netherlands, measuring (angular) velocity with a sampling rate of 104 samples per second. The ranges of the accelerometer and gyroscope were set to ± 4 m/s^2^ and ± 500°/s, respectively.

While performing the sitting tests (SIT and SIT UNSTABLE), one inertial measurement unit was placed at the estimated height of the participant's center of mass, thus on the upper back at the height of T7. An elastic band was used to hold the inertial measurement unit in place during the tests. For both sitting tests a height adjustable treatment table was used. The SIT UNSTABLE task was performed with a round, inflatable balance cushion (MamboMax, 45 cm, filled: semi-soft) that was placed on the treatment table. For the standing tests (STAND, STAND EC, STAND FOAM), the inertial measurement unit was placed on the lower back at the height of L5/S1. For the STAND FOAM test the participant stood on a foam balance pad (Airex balance pad, 50 × 41 × 6 cm). A measurement was excluded if the participant was unable to perform the test, made a corrective step, used or needed hand support, was visibly distracted, or in case of an observable clonus. To avoid unwanted movements during the sitting and standing tests, participants were instructed to stay still as possible and to refrain from talking.

During the 2-min walking test (2MWT), participants were equipped with two extra inertial measurement units on top of the left and right foot, with the range set ± 8 m/s^2^ and ± 500°/s. Participants walked for two minutes on a 14-m walking path with cones at both ends. They were instructed to walk on a self-selected comfortable walking speed. The data of participants with an average walking speed of ≤0.05 m/s for 2 min were excluded for analysis, to ensure that gait variables were measured reliable. Participants who required a walking aid, performed a 2MWT with walking aid (2MWT_with_). If the participant was able to, a 2MWT without walking aid (2MWT_without_) was performed. Before and after each walking test, participants stood still for five seconds to enable an accurate start and end of the test. A measurement was excluded if the participant stopped walking, was visibly distracted, or in case of an observable clonus. Participants were instructed to refrain from talking. In case of all tests, participants were allowed to retry the test in case of a faulty measurement and they were allowed to rest before starting the following test.

### Data process

An extensive description of the data processing and calculation of the balance and gait variables measured with inertial measurement units while sitting, standing, and walking is provided by Felius et al.^9,12^. During the sitting and standing tests, the inertial measurement unit-measured postural sway of the body. The total postural sway trajectory during the test was captured by the variable “path” in mm/s^4^ with good to excellent reliability.^9^ A greater path score indicates more postural sway movement, suggesting the need for more corrective movements, which is associated with a poorer performance.^17,18^ Processing the data exposed that, in case of the sitting and standing tests, patients with low path scores (i.e., good balance) were not discriminated from patients with high path scores (i.e., bad balance). Therefore, we excluded patients with a good balance, determined by a BBS score of ≥ 45, from further analysis. An extensive explanation of this is described in Supplementary material 1.

For the walking tests, three variables measured with inertial measurement units were used for further analysis to describe the way the patient walks: tempo, symmetry, and postural stability. These variables are obtained from a principal component analysis performed by Felius et al.^13^ In total, one to maximal 11 variables were obtained during performing the tests with inertial measurement units (one for each balance test, three for each walking test).

### Statistical analysis

The current study has a cross-sectional design since we aim to explore the added value of sensor-based tests over conventional measures in explaining the variance in walking and ADL independency in patients during inpatient stroke rehabilitation. We analyzed the cross-sectional data measured within the first week after admission to the rehabilitation center. First, a descriptive analysis of the participant's clinical and demographical characteristics at admission to the rehabilitation center was undertaken. Evaluating the association of each independent variable (both conventional measures and inertial measurement unit-measured variables) with both dependent variables (i.e., BI and FAC scores) was done by univariable linear regression analyses. The association between the independent and dependent variable was demonstrated by the squared correlation coefficient (R^2^). The higher the R^2^, the stronger the association between both variables.

Secondly, hierarchical multivariable regression analyses were conducted to explore the added value of inertial measurement units besides the conventional measures MI, TCT, and BBS in explaining the variance in the BI and FAC (dependent variables). Small sample sizes and missing data required us to account for statistical power. Therefore, we opted to conduct hierarchical multivariable regression analyses with two independent variables entered into two blocks. The first block included one conventional measure (i.e., MI, TCT, or BBS) and in the second block one of the variables measured by the inertial measurement unit was added. This was repeated for all combinations of conventional measures and inertial measurement unit-measured variables. When the second model (conventional + inertial measurement unit-measured variable) explained the variance in the BI or FAC significantly (i.e., *R*^2^ with *p*-value ≤ .05), the change statistics were consulted. Using the *R*-square change (Δ*R*^2^), the incremental contribution of the inertial measurement unit between model 1 and model 2 could be determined.^19^ The *R*-square change was tested with an *F*-test, that is, the *F*-change or Δ*F*, to determine the significance of the *R*-square change. With a significant *F*-change (*p*  ≤ .05), the variable added to the model significantly improved the model prediction. In other words, the added value of that particular variable measured with an inertial measurement unit on top of a conventional test was confirmed. When the added value of a variable was shown, also the standardized beta coefficients were examined. A positive beta coefficient demonstrates a positive association between the independent variable and the dependent variable, and for a negative beta coefficient applies the opposite.

Listwise deletion was used to exclude participants who has at least one missing value of the specified independent variables. All analyses were conducted using the Statistical Package for Social Sciences software version 28.0.1.0 (SPSS Inc., Chicago, IL, USA). Data are available from the corresponding author.

## Results

In total, 115 consecutive patients with stroke enroled in our study. The included participants were admitted to the rehabilitation center on average 23.73 days poststroke (ranging from 5 days to 96 days poststroke). 45.2% of the patients were mildly disabled or fully ADL independent (BI ≥ 15).^20^ About half of the patients (50.4%) were able to walk independently (FAC ≥ 4) at admission to the rehabilitation center, mainly using a walking aid (62.6%). 14.8% of the patients with stroke were not able to walk at all (FAC = 0). In Table 2, the patient characteristics are shown. Missing data in the conventional measures were caused by time constraints imposed by the therapist during rehabilitation. Furthermore, a significant portion of the data collected by the inertial measurement units was excluded in patients with a BBS score ≥ 45, as outlined in Supplementary material 1. Besides that, three other reasons resulted in missing inertial measurement unit-based data: technical issues with the device, the inability of a patient to perform the test, or one of the exclusion criteria as described in the methods section. Therefore, sample sizes varied for each test. Patient characteristics of each sample performing each test using inertial measurement units, are outlined in Supplementary material 2.

**Table 2.** Patient characteristics.

	*N*	Mean ± SD	Median [IQR]
Age	115	72.25 ± 12.34	74 [64, 82]
Gender (male)		54.8%	
Days poststroke to admission		23.73 ± 14.91	20 [15, 29]
Stroke type			
Ischemic		80.0%	
Hemorrhagic		15.7%	
SAB		4.3%	
Affected side			
Left		44.3%	
Right		31.3%	
None		7.8%	
Both sides		3.5%	
Other		13.0%	
BI	111	13.32 ± 4.97	13 [10, 18]
ADL dependent (BI ≤ 14)		54.8%	
FAC	115	3.02 ± 1.63	4 [2, 4]
Dependent walking (FAC 0/1/2/3)	17/4/17/19	49.6%	
Independent walking (FAC 4/5)	38/20	50.4%	
MI	105	79.6 ± 24.43	91 [72, 100]
TCT	113	88.58 ± 22.06	100 [87, 100]
BBS	109	36.59 ± 16.82	41 [26, 51]
SIT path^a^	58	21.01 ± 8.84	
SIT FOAM path^a^	51	30.93 ± 15.00	
STAND path^a^	43	40.62 ± 21.78	
STAND EC path^a^	35	30.28 ± 21.22	
STAND FOAM path^a^	25	29.04 ± 15.39	
2MWT with tempo	45	−3.41 ± 2.95	
2MWT with asymmetry	45	−.47 ± 1.29	
2MWT with postural stability	45	.00 ± 1.41	
2MWT without tempo	37	3.86 ± 4.79	
2MWT without symmetry	36	−.68 ± 1.04	
2MWT without postural Stability	37	−.23 ± .86	

IQR = interquartile range; SAB = subarachnoid hemorrhage; BI = Barthel Index; ADL = activities of daily living; FAC = Functional Ambulation Categories; MI = Motricity Index; TCT = Trunk Control Test; BBS = Berg Balance Scale; see Table 1 for explanation of each inertial measurement unit-based test; see Supplementary material 2 for patient characteristics of each sample.

a Stroke sample selected based on BBS < 45, see Supplementary material 1.

### Association with clinically relevant outcome measures

Results of the univariable linear regression analyses are shown in Table 3. The BBS explained most variance of the BI (*R*^2^ = .527, *p* < .001). As balance improved, the independency in ADL increased [β = .726, *t*(104) = 10.762]. When specifically looking at the tests measured by inertial measurement units, the postural sway measured by an inertial measurement unit while standing on foam explained most variance of the BI (*R*^2^ = .200, *p* = .033). As postural sway, measured while standing on foam, decreased, the independency in ADL increased [β = −.447, *t*(21) = −2.288].

**Table 3.** Univariable regression analyses.

Independent variables	Model statistics: ADL independency (BI)		Model statistics: Walking ability (FAC)	
MI	*R*^2^ = .325; *F*(1.100) = 48.155; *p* < .001*	β = .570; *t*(100) = 6.939; *p* < .001*	*R*^2^ = .511; *F*(1.103) = 107.438; *p* < .001*	β = .715; *t*(103) = 10.365; *p* < .001*
TCT	*R*^2^ = .380; *F*(1.107) = 65.565; *p* < .001*	β = .616; *t*(107) = 8.097; *p* < .001*	*R*^2^ = .436; *F*(1.111) = 85.763; *p* < .001*	β = .660; *t*(111) = 9.261; *p* < .001*
BBS	*R*^2^ = .527; *F*(1.104) = 115.811; *p* < .001*	β = .726; *t*(104) = 10.762; *p* < . .001*	*R*^2^ = .727; *F*(1.107) = 284.348; *p* < .001*	β = .852; *t*(107) = 16.863; *p* < .001*
SIT path	*R*^2^ = .059; *F*(1.53) = 3.302; *p* = .075		*R*^2^ = .013; *F*(1.56) = .760; *p* = .387	
SIT FOAM path	*R*^2^ = .087; *F*(1.46) = 4.360; *p* = .042*	β = −.294; *t*(46) = −2.088; *p* = .042	*R*^2^ = .023; *F*(1.49) = 1.137; *p* = .292	
STAND path	*R*^2^ = .065; *F*(1.38) = 2.662; *p* = .111		*R*^2^ = .066; *F*(1.41) = 2.885; *p* = .097	
STAND EC path	*R*^2^ = .039; *F*(1.30) = 1.221; *p* = .278		*R*^2^ = .043; *F*(1.33) = 1.470; *p* = .234	
STAND FOAM path	*R*^2^ = .200; *F*(1.21) = 5.234; *p* = .033*	β = −.447; *t*(21) = −2.288; *p* = .033	*R*^2^ = .125; *F*(1.23) = 3.281; *p* = .083	
2MWT with tempo	*R*^2^ = .165; *F*(1.40) = 7.901; *p* = .008*	β = .406; *t*(40) = 2.812; *p* = .008*	*R*^2^ = .207; *F*(1.43) = 11.231; *p* = .002*	β = .455; *t*(43) = 3.351; *p* = .002*
2MWT with symmetry	*R*^2^ = .001; *F*(1.40) = .027; *p* = .870		*R*^2^ = .068; *F*(1.43) = 3.158; *p* = .083	
2MWT with postural stability	*R*^2^ = .007; *F*(1.40) = .296; *p* = .590		*R*^2^ = .000; *F*(1.43) = .002; *p* = .968	
2MWT without tempo	*R*^2^ = .052; *F*(1.34) = 1.849; *p* = .183		*R*^2^ = .141; *F*(1.35) = 5.764; *p* = .022*	β = .376; *t*(35) = 2.401; *p* = .022*
2MWT without symmetry	*R*^2^ = .110; *F*(1.33) = 4.093; *p* = .051		*R*^2^ = .015; *F*(1.34) = .510; *p* = .480	
2MWT without postural stability	*R*^2^ = .000; *F*(1.34) = .013; *p* = .911		*R*^2^ = .004; *F*(1.35) = .142; *p* = .708	

BI = Barthel Index; FAC = Functional Ambulation Categories; MI = Motricity Index; TCT = Trunk Control Test; BBS = Berg Balance Scale; see Table 1 for explanation of each inertial measurement unit-based test. *p ≤ .05.

The BBS also explained most variance of the FAC score (*R*^2^ = .727, *p* < .001), indicating as balance improved, the ability of walking independent also increased [β = .852; *t*(107) = 16.863]. When looking at the tests measured by inertial measurement units, tempo measured during the 2MWT with walking aid, explained most variance of the FAC (*R*^2^ = .207, *p* = .002). As walking speed during this 2MWT improved, the ability of walking independent increased [β = .455; *t*(43) = 3.351].

### Added value of inertial measurement units

Supplementary material 3 shows the results of the hierarchical multivariable regression analyses with which we explored the added value of inertial measurement units in explaining the variance in ADL independency on top of conventional measures. Three of the 33 models consisting two variables, showed a significant *F*-change by adding an inertial measurement unit-measured variable. Model statistics are shown in Table 4. The multivariable analyses showed that symmetry measured during the 2MWT without walking aid, exhibited the most substantial contribution (ΔR^2^ = 18.6%, *p* = .013) among all inertial measurement unit-measured variables in explaining the variance in the ADL independency. As post-stroke asymmetry decreases, independence in ADL improves (β = .438, *p* = .013). Besides that, we determined a statistically significant and positive change by adding tempo measured during the 2MWT with walking (β = .340, *p* = .037) to the model with the MI, resulting in a model explaining 12.2% of the variance in BI. The addition of tempo measured during the 2MWT_with_ to a model with TCT revealed that tempo has a significant and positive impact on BI (β = .375, *p* = .014), resulting in a model explaining 15.4% of the variance in BI. As walking speed increases, the independence in ADL improves. Regarding models involving the BBS, the addition of a variable measured with an inertial measurement unit did not result in a statistically significant change in explaining the variance in BI.

**Table 4.** Statistics of the hierarchical multivariable regression models in which the added value of the inertial measurement unit-measured variable is significant in explaining ADL independency and walking ability.

	Model variables	Statistics model 2
Models explaining variance in ADL independency (BI)	MI + 2MWT with Tempo	Adj. R^2^ = .122; *F*(2.35) = 3.570; *p* = .039*
	MI + 2MWT without symmetry	Adj. *R*^2^ = .157; *F*(2.30) = 3.983; *p* = .029*
	TCT + 2MWT with tempo	Adj. *R*^2^ = .154; *F*(2.38) = 4.653; *p* = .016*
Models explaining variance in walking ability (FAC)	MI + 2MWT with tempo	Adj. *R*^2^ = .233; *F*(2.38) = 7.081; *p* = .002*
	MI + 2MWT without tempo	Adj. *R*^2^ = .123; *F*(2.32) = 3.388; *p* = .046*
	TCT, 2MWT with tempo	Adj. *R*^2^ = .170; *F*(2.41) = 5.401; *p* = .008*

BI = Barthel Index; FAC = Functional Ambulation Categories; MI = Motricity Index; TCT = Trunk Control Test; see Table 1 for explanation of each inertial measurement unit-based test. *p ≤ .05.

The results of the hierarchical multivariable regression models explaining the variance in walking independently are shown in Supplementary material 4. Three of the 33 models consisting two variables, showed a significant F-change by adding an inertial measurement unit-measured variable. Table 4 shows the model statistics. When tempo measured during the 2MWT with and without walking aid was added (separately) to a model including the MI, this inertial measurement unit-measured variable demonstrated a statistically significant change, resulting in a model explaining 23.3% and 12.3% of the variance in FAC score. Both variables showed a significant and positive effect on the FAC score (β = .455, *p* = .003 and β = .423, *p* = .015, respectively). The addition of tempo measured during the 2MWT with walking aid to a model with TCT, also increased the proportion of explained variance in the FAC score with a R-square change of 18.3%. A significant positive impact of tempo on the FAC score was shown (β = .429, *p* = .004). In all cases applied: when the walking speed during the 2MWT increases, the ability to walk independent improves. Regarding models involving the BBS, the addition of a variable measured with an inertial measurement unit did not result in a statistically significant change in explaining the variance in FAC.

## Discussion

In this cross-sectional study, we explored whether inertial measurement units are of added value in explaining the variance in the independency of ADL (BI) and walking ability (FAC) after stroke, on top of the conventional tests MI, TCT and BBS. Although we found univariable associations between postural sway and both the BI and FAC scores, in multivariable analyses postural sway measured with an inertial measurement unit showed no added value in explaining independency in ADL and walking ability in patients after stroke. Therefore, we reject our hypothesis that inertial measurement units would add predictive value beyond conventional tests in explaining variance in these outcome measures.

This finding is inconsistent with former research which demonstrated that sitting balance was an early indicator of ADL independence^21^ and postural control while standing was strongly correlated with both walking performance and ADL independency in patients with stroke.^22^ However, in the current study, conventional tests alone explained most of the variance in both ADL independency and walking ability. These findings may be attributable to the different constructs that the conventional and inertial measurement units are measuring. Conventional tests determine whether the patient can perform certain (balance) tasks, whereas inertial measurement units allow for an objective assessment of how and why the patient's balance and/or gait is impaired. The presence of these different constructs is exemplified by the weak associations we found between the postural sway during sitting and standing and the BBS scores (see Supplementary material 1), which is consistent with previous studies showing that postural sway was barely associated with the TCT and BBS.^23,24^

Tempo and symmetry measured with inertial measurement units during walking were identified as statistically significant contributors to a model with either the MI and TCT in explaining the variance in ADL independency and walking ability. Multivariable analyses showed that symmetry measured during the 2MWT without walking aid, exhibited the most substantial contribution in explaining the variance in the ADL independency. As poststroke asymmetry decreases, independence in ADL improves. Gait symmetry is known to be related to balance and walking speed,^25,26^ which is subsequently correlated to ADL independency, as measured by the BI and Functional Independence Measure (FIM).^27,28^ Furthermore, walking speed assessed during the 2MWT with a walking aid, has emerged as the primary contributor to explaining the variance in walking ability. As tempo during walking increases, walking independence improves, which is consistent with former studies.^29,30^ However, it should be noted that an increase in walking speed does not necessarily indicate improved gait quality, as it may compromise safety, increase fatigue, and fall risk.

The added value of the variables measured with inertial measurement units in explaining variance in independency in ADL and walking was scant. Univariable regression analyses revealed that each conventional measure had a stronger individual association with the outcome measures. The BBS explained 52.7% and 72.7% of the variance in ADL independency and independent walking, respectively, with no improvement from adding inertial measurement unit-measured variables. These findings support the adequacy of conventional tests in explaining the variance of both outcome measures. Conventional measures assess a distinct construct compared to the variables measured by inertial measurement units, which could be elucidated within the framework of the ICF-model. Conventional measures predominantly evaluate “Activities”, while inertial measurement unit-measured variables are rooted in the “Body functions and structures” level of the ICF.^6^ Overly focusing on the distinctive variables measured by inertial measurement units oversimplifies the influence of various factors on independence in ADL and walking ability in daily life.

The current study has several limitations that we aim to address. The first methodological limitation, is the cross-sectional design of the study. Due to insufficient longitudinal data, we were assigned to explore this with cross-sectional data measured within the first week after admission to the rehabilitation center. Although we did not study this longitudinally, based on the current results is the added value of inertial measurement units in predicting the long-term recovery after stroke not expected. Conventional tests appear to sufficiently explain the proportional variance in ADL independency and independent walking and variables measured by inertial measurement units are not of incremental contribution to this.

The second limitation was the inclusion of only measures determining the motor function after stroke. Including other variables, such as stroke severity, urinary continence, age, and sex, in the regression models could improve the explanation of variance in ADL and walking independence. Previous research identified these factors as prognostic in predicting ADL independence and independent walking after stroke.^14,31–33^ In light of these findings, it is evident that the incorporation of distinct variables beyond the conventional tests determining motor function, might have enriched the proportion explained variance in the outcome measures.

The added value of variables measured with an inertial measurement unit is limited in explaining variance in ADL independence and walking ability. Especially tempo measured with inertial measurement units during walking was identified as statistically significant and positive contributor to a model with either the MI and TCT in explaining the variance in ADL independency and walking ability. However, it is worth considering whether the use of inertial measurement units is essential for this purpose, as walking speed can also be measured using a general stopwatch, eliminating the need for an additional device. These findings enhance the understanding of using inertial measurement units during stroke rehabilitation and highlight the caution needed when applying inertial measurement units to predict physical recovery after stroke.

Lastly, although 115 participants were initially included, the sample sizes varied considerably across tests from 25 to 58. This resulted in relatively homogeneous outcome distributions, limiting the potential for high prediction accuracy. On the one hand, although we excluded patients with a BBS score of ≥ 45, no discrimination was possible between moderately to well-performing stroke survivors during less challenging tests (see Supplementary material 1). On the other hand, the more demanding tests were completed by fewer participants, resulting in small sample sizes. We underestimated the impact of this natural selection within the target population. Conventional clinical tests like the BBS inherently account for this by design. However, such adaptive structuring is not yet feasible in inertial measurement unit-based tests. As a result, the outcomes measured with inertial measurement units showed limited variability, which inherently restricts the prediction accuracy that can be achieved.

**Clinical messages**

- The added value of postural sway and gait variables measured with an inertial measurement unit is limited in explaining variance in independence in activities of daily living and independent walking.
- The findings highlight the caution needed when applying inertial measurement units to predict recovery after stroke.

## Supplemental Material

sj-docx-1-cre-10.1177_02692155251362742 - Supplemental material for The added value of sensor-based tests in explaining the variance in walking and ADL independency after stroke: An exploratory study Supplemental material, sj-docx-1-cre-10.1177_02692155251362742 for The added value of sensor-based tests in explaining the variance in walking and ADL independency after stroke: An exploratory study by Natasja Charon Wouda, Marieke Geerars, Martijn Frits Pisters, Johanna Maria Augusta Visser-Meily and Michiel Punt in Clinical Rehabilitation

sj-docx-2-cre-10.1177_02692155251362742 - Supplemental material for The added value of sensor-based tests in explaining the variance in walking and ADL independency after stroke: An exploratory study Supplemental material, sj-docx-2-cre-10.1177_02692155251362742 for The added value of sensor-based tests in explaining the variance in walking and ADL independency after stroke: An exploratory study by Natasja Charon Wouda, Marieke Geerars, Martijn Frits Pisters, Johanna Maria Augusta Visser-Meily and Michiel Punt in Clinical Rehabilitation

sj-docx-3-cre-10.1177_02692155251362742 - Supplemental material for The added value of sensor-based tests in explaining the variance in walking and ADL independency after stroke: An exploratory study Supplemental material, sj-docx-3-cre-10.1177_02692155251362742 for The added value of sensor-based tests in explaining the variance in walking and ADL independency after stroke: An exploratory study by Natasja Charon Wouda, Marieke Geerars, Martijn Frits Pisters, Johanna Maria Augusta Visser-Meily and Michiel Punt in Clinical Rehabilitation

sj-docx-4-cre-10.1177_02692155251362742 - Supplemental material for The added value of sensor-based tests in explaining the variance in walking and ADL independency after stroke: An exploratory study Supplemental material, sj-docx-4-cre-10.1177_02692155251362742 for The added value of sensor-based tests in explaining the variance in walking and ADL independency after stroke: An exploratory study by Natasja Charon Wouda, Marieke Geerars, Martijn Frits Pisters, Johanna Maria Augusta Visser-Meily and Michiel Punt in Clinical Rehabilitation

sj-docx-5-cre-10.1177_02692155251362742 - Supplemental material for The added value of sensor-based tests in explaining the variance in walking and ADL independency after stroke: An exploratory study Supplemental material, sj-docx-5-cre-10.1177_02692155251362742 for The added value of sensor-based tests in explaining the variance in walking and ADL independency after stroke: An exploratory study by Natasja Charon Wouda, Marieke Geerars, Martijn Frits Pisters, Johanna Maria Augusta Visser-Meily and Michiel Punt in Clinical Rehabilitation
