# Supplementary material for: The added value of sensor-based tests in explaining the variance in walking and ADL independency after stroke: An exploratory study
Source: Clin Rehabil. 2025 Aug 3;39(10):1366–77. doi: 10.1177/02692155251362742 (PMC12414103; doi:10.1177/02692155251362742)
Supplement: sj-docx-2-cre-10.1177_02692155251362742 - Supplemental material for The added value of sensor-based tests in explaining the variance in walking and ADL independency after stroke: An exploratory study [file sj-docx-2-cre-10.1177_02692155251362742.docx]

**Supplementary material 1.**

The stroke population is heterogeneous as a result of which certain variables measured with inertial measurement units, may not be suitable to discriminate patients with low postural sway scores (i.e., good balance) from patients with high scores (i.e., bad balance). If a variable appears to be not sensitive to differences across (a certain group of) the stroke population, then we could consider excluding this specific group or test from further analysis. Therefore, identifying relevant inertial measurement unit-measured variables was the basis for further exploration on the added value of inertial measurement units besides conventional tests. Relevant inertial measurement unit-based tests were identified by visualizing whether an inertial measurement unit-based test was performed by patients with a certain Berg Balance Scale (BBS) score in a scatterplot, see Figure 1 and Figure 2. Patients with lower BBS scores, were less likely to perform the difficult inertial measurement unit-based test such as standing with eyes closed or standing on foam and walking. We called this the natural selection.

The color coded cross in Figure 1 and 2 represent the average of the standardized (z-scored) inertial measurement unit-measured data per BBS score of that particular variable. In this way, the color codes gain insight in the variability of the inertial measurement unit-measured data of the patients with stroke, and therefore, whether the inertial measurement unit-measured test was able to discriminate patients with low scores from patients with high scores. In Figure 1 the sitting and standing tasks were shown, in which the crosses of the patients with higher BBS scores were mainly colored in blue. This indicates that all mean z-scores were below zero, reflecting a minimal postural sway during the balance task measured by an inertial measurement unit. With the inertial measurement unit-measured data measured during the sitting and standing tests, we are not able to discriminate patients with low scores (i.e. good balance, high BBS scores) from patients with high scores (i.e. bad balance, low BBS scores). Since it measures limited variability in the inertial measurement unit-measured data of patients with stroke while the BBS scores become higher (i.e. the crosses are the same shades of blue for patients with higher BBS scores), we excluded patients with a BBS score of ≥ 45 from further analysis.

Figure 2 shows the ability of patients to walking for 2 minutes with or without a walking aid and the corresponding BBS score. The color coded crosses showed large differences across this group of stroke patients. Since this variability proved the ability of discriminating patients with low scores from patients with high scores, no exclusion was needed and the stroke sample remained the same.

In Table 5 we have presented data concerning the measures of dispersion and correlation relationship of the inertial measurement unit-based tests and BBS scores on group level. The (interquartile) ranges demonstrate that excluding patients with BBS ≥ 45 from the balance tasks, has little to no impact on the dataset. As a result of excluding these patients, a smaller sample remains. This affects the distribution in the BBS scores (from 0 to 44 instead of 56) and therefore the correlational relationship with the BBS. This was shown on group level using Pearson’s correlation coefficient (r). Tempo measured while walking with a walking aid, correlated the most, but still moderate, with the BBS (r = .538, p < .001). When comparing the original dataset and customized dataset, overall Pearson’s r decreases and not all variables show a significant correlation with the BBS.

Patient characteristics and clinical outcomes of each sample are shown in Supplementary material 2.

**Figure 1.** The BBS score plotted against the ability of each patient to perform the consecutive balance tests measured by an inertial measurement unit


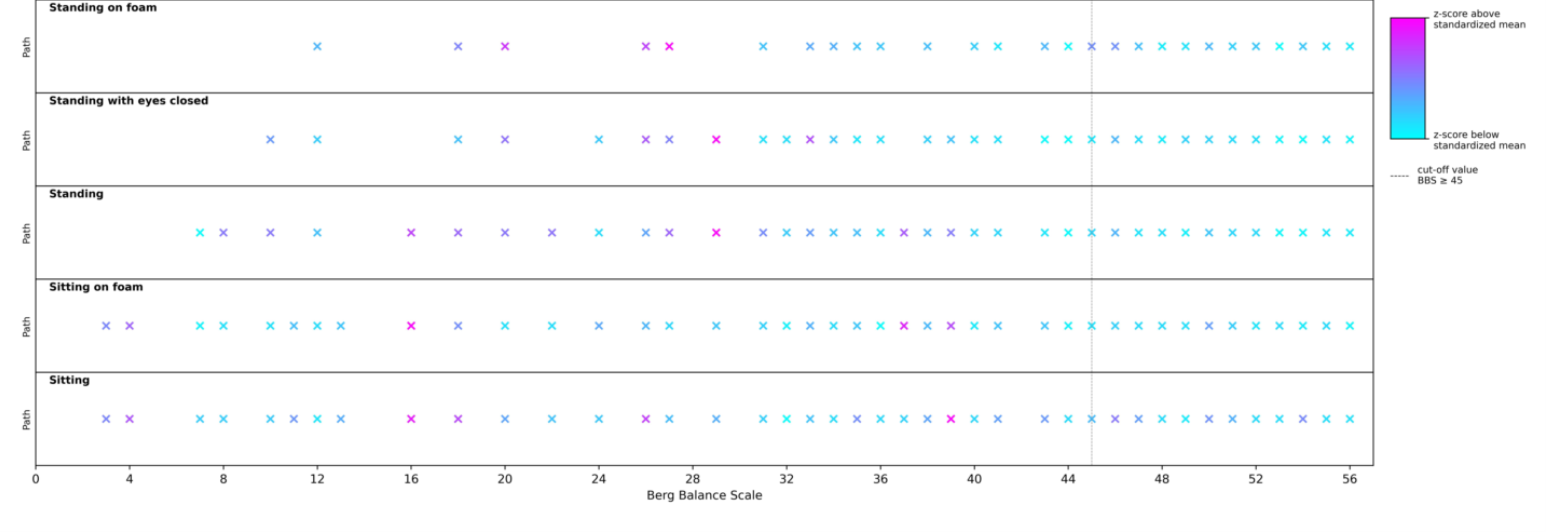


**Figure 2.** The BBS score plotted against the ability of each patient to perform the consecutive walking tests measured by inertial measurement units

**
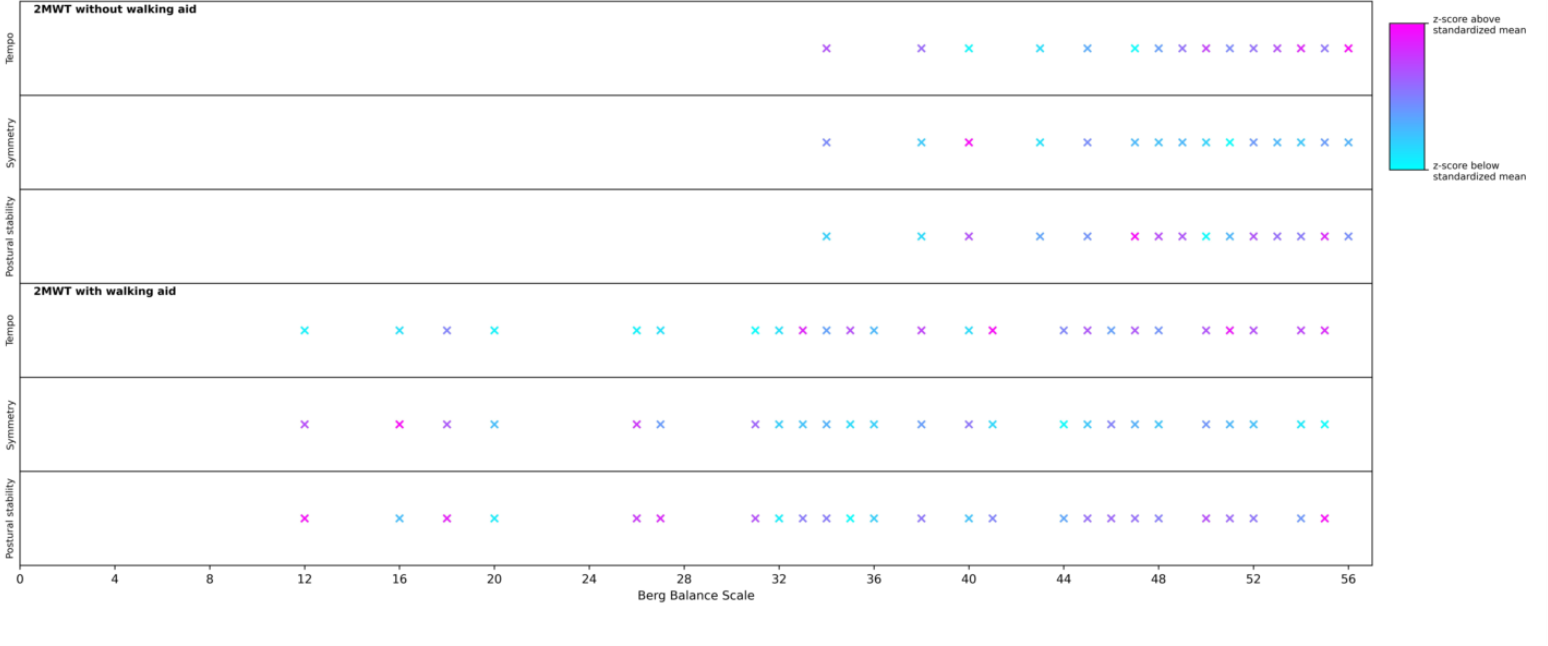
**

**Table 5.** Distribution of variables measured during tests^#^ by inertial measurement units in median [IQR] and Pearson's correlation coefficient (p-value)

|  | **Original dataset** | **N** | **Customized dataset** | **N** |
| --- | --- | --- | --- | --- |
| SIT Path | -.31 [-.66,.24] | 109 | -.33 [-.67,.24] | 58 |
|  | -.304 (.001) * |  | -.285 (.030) * |  |
| SIT FOAM Path | -.21 [-.58,.22] | 102 | -.24 [-.63,.33] | 51 |
|  | -.300 (.002) * |  | -.145 (.312) |  |
| STAND Path | -.29 [-.70,.27] | 94 | -.30 [-.71,.46] | 43 |
|  | -.489 (< .001) * |  | -.188 (.227) |  |
| STAND EC Path | -.28 [-.56,.11] | 86 | -.42 [-.60,.24] | 35 |
|  | -.450 (< .001) * |  | -.264 (.126) |  |
| STAND FOAM Path | -.14 [-.69,.25] | 75 | -.33 [-.56,.15] | 25 |
|  | -.436 (< .001) * |  | -.413 (.040) * |  |
| 2MWTwith |  |  |  |  |
| Tempo | -.02 [-.86,.64] | 45 |  |  |
|  | .538 (< .001) * |  |  |  |
| Symmetry | -.11 [-.70,.53] | 45 |  |  |
|  | -.445 (.002) * |  |  |  |
| Postural stabiliy | -.16 [-.59,.85] | 45 |  |  |
|  | -.068 (.657) |  |  |  |
| 2MWTwithout |  |  |  |  |
| Tempo | -.02 [ -.71,.62] | 37 |  |  |
|  | .442 (.007) * |  |  |  |
| Symmetry | -.21 [ -.82,.59] | 37 |  |  |
|  | -.151 (.388) |  |  |  |
| Postural stabiliy | .12 [-.92,.70] | 37 |  |  |
|  | .182 (.289) |  |  |  |
| ^#^*see table 1 for explanation of each inertial measurement unit-based test* | | | | |
