# Supplementary material for: The added value of sensor-based tests in explaining the variance in walking and ADL independency after stroke: An exploratory study
Source: Clin Rehabil. 2025 Aug 3;39(10):1366–77. doi: 10.1177/02692155251362742 (PMC12414103; doi:10.1177/02692155251362742)
Supplement: sj-docx-3-cre-10.1177_02692155251362742 - Supplemental material for The added value of sensor-based tests in explaining the variance in walking and ADL independency after stroke: An exploratory study [file sj-docx-3-cre-10.1177_02692155251362742.docx]

#### **Supplementary material 2.**

Table 6. Patient characteristics per sample performing each inertial measurement unit-measured test

| **Patient characteristics** | **Total sample** | **SIT Path *** | **SIT FOAM Path *** | **STAND Path *** | **STAND EC Path *** | **STAND FOAM Path *** | **2MWT with aid** | | | **2MWT without aid** | | |
| --- | --- | --- | --- | --- | --- | --- | --- | --- | --- | --- | --- | --- |
|  |  |  |  |  |  |  | **Tempo** | **Symmetry** | **Postural stability** | **Tempo** | **Symmetry** | **Postural stability** |
| mean ± SD | N/A | 21.01 ± 8.84 | 30.93 ± 15.00 | 40.62 ± 21.78 | 30.28 ± 21.22 | 29.04 ± 15.39 | -3.41 ± 2.95 | -.47 ± 1.29 | .00 ± 1.41 | 3.86 ± 4.79 | -.68 ± 1.04 | -.23 ± .86 |
| N | 115 | 58 | 51 | 43 | 35 | 25 | 45 | | | 37 | | |
| Age | 72.25 ± 12.34 | 75.03 ± 12.76 | 74.76 ± 13.16 | 75.67 ± 13.72 | 77.03 ± 13.18 | 78.36 ± 10.22 | 76.98 ± 12.00 | | | 67.51 ± 12.33 | | |
| Gender (male) | 54.8% | 48.3% | 51.0% | 48.8% | 48.6% | 48.0% | 48.9% | | | 62.2% | | |
| Days poststroke to admission | 23.73 ± 14.91 | 23.57 ± 13.92 | 21.84 ± 12.55 | 19.74 ± 9.29 | 20.03 ± 9.58 | 20.76 ± 10.52 | 20.00 ± 11.79 | | | 23.49 ± 10.68 | | |
| Stroke type  ischemic  hemorrhagic  SAB | 80.0%  15.7%  4.3% | 75.9%  20.7%  3.4% | 72.5%  23.5%  3.9% | 74.4%  20.9%  4.7% | 77.1%  17.1%  5.7% | 84.0%  12.0%  4.0% | 84.4%  11.1%  4.4% | | | 83.8%  10.8%  5.4% | | |
| Affected side  left  right  none  both sides  other | 44.3%  31.3%  7.8%  3.5%  13.0% | 48.3%  32.8%  1.7%  6.9%  10.3% | 47.1%  33.3%  2.0%  5.9%  11.8% | 44.2%  37.2%  0%  4.7%  14.0% | 45.7%  37.1%  0%  0%  17.1% | 52.0%  32.0%  0%  0%  16.0% | 46.7%  31.1%  0%  0%  22.2% | | | 32.4%  32.4%  21.6%  0%  13.5% | | |
| BI | 13.32 ± 4.97 | 10.69 ± 4.41 | 11.63 ± 3.69 | 12.20 ± 3.12 | 12.53 ± 3.06 | 12.26 ± 3.14 | 13.90 ± 3.66 | | | 17.17 ± 3.19 | | |
| ADL dependent (BI ≤ 14) | 54.8% | 80.0% | 77.1% | 75.0% | 65.7% | 76.0% | 48.9% | | | 16.2% | | |
| FAC | 3.02 ± 1.63 | 2.21 ± 1.51 | 2.47 ± 1.41 | 2.72 ± 1.22 | 3.00 ± 1.085 | 2.88 ± 1.17 | 3.38 ± .912 | | | 4.38 ± .639 | | |
| dependent walking (FAC<4) | 49.6% | 74.1% | 70.6% | 67.4% | 60.0% | 64.0% | 46.7% | | | 8.1% | | |
| MI | 79.6 ± 24.43 | 70.75 ± 25.51 | 75.02 ± 23.77 | 78.75 ± 22.21 | 82.22 ± 21.71 | 82.57 ± 23.68 | 88.71 ± 14.61 | | | 93.26 ± 8.93 | | |
| TCT | 88.58 ± 22.06 | 85.87 ± 21.11 | 89.82 ± 17.49 | 92.69 ± 15.46 | 93.59 ± 16.10 | 93.36 ± 17.27 | 94.48 ± 15.86 | | | 98.46 ± 5.48 | | |
| BBS * | 36.59 ± 16.82 | 24.02 ± 13.50 | 26.82 ± 11.88 | 28.95 ± 10.05 | 31.00 ± 8.63 | 32.40 ± 7.86 | 38.51 ± 11.14 | | | 50.67 ± 5.26 | | |
| ** Stroke sample selected based on BBS<45, see Supplementary material 1; See table 1 for explanation of each inertial measurement unit-based test; SAB = subarachnoid hemorrhage; BI = Barthel Index; ADL = activities of daily living; FAC = Functional Ambulation Categories; MI = Motricity Index; TCT = Trunk Control Test; BBS = Berg Balance Scale; N/A = not applicable; 2MWT = 2 minutes walking test* | | | | | | | | | | | | |
