# Supplementary material for: The added value of sensor-based tests in explaining the variance in walking and ADL independency after stroke: An exploratory study
Source: Clin Rehabil. 2025 Aug 3;39(10):1366–77. doi: 10.1177/02692155251362742 (PMC12414103; doi:10.1177/02692155251362742)
Supplement: sj-docx-4-cre-10.1177_02692155251362742 - Supplemental material for The added value of sensor-based tests in explaining the variance in walking and ADL independency after stroke: An exploratory study [file sj-docx-4-cre-10.1177_02692155251362742.docx]

#### **Supplementary material 3.**

Table 7. Multivariable regression analyses to explore the added value of inertial measurement units-measured tests besides the conventional measures MI, TCT and BBS in explaining the variance in ADL independency (determined by the Barthel Index)

|  | **Model statistics** |  |  |
| --- | --- | --- | --- |
| **Independent variables** | **Model 1 (conventional test)** | **Model 2 (conventional + inertial measurement unit-measured variable)** | **Change statistiscs model 2** |
| MI, SIT Path | R^2^ = .276; F(1.48) = 18.274; p < .001* | Adj. R^2^ = .287; F(2.47) = 10.842; p < .001* | ∆R^2^ = .040; ∆F(1.47) = 2.746; p = .104 |
| MI, SIT FOAM Path | R^2^ = .155; F(1.41) = 7.536; p = .009* | Adj. R^2^ = .171; F(2.40) = 5.341; p = .009* | ∆R^2^ = .056; ∆F(1.40) = 2.813; p =.101 |
| MI, STAND Path | R^2^ = .129; F(1.35) = 5.200; p = .029* | Adj. R^2^ = .093; F(2.34) = 2.840; p = .072 |  |
| MI, STAND EC Path | R^2^ = .109; F(1.27) = 3.320; p = .080 | Adj. R^2^ = .056; F(2.26) = 1.825; p = .181 |  |
| MI, STAND FOAM Path | R^2^ = .316; F(1.19) = 8.779; p = .008* | Adj. R^2^ = .343; F(2.18) = 6.230; p = .009* | ∆R^2^ = .093; ∆F(1.18) = 2.833; p = .110 |
| MI, 2MWTwith Tempo | R^2^ = .058; F(1.36) = 2.214; p = .145 | Adj. R^2^ = .122; F(2.35) = 3.570; p = .039* | ∆R^2^ = .112; ∆F(1.35) = 4.699; p = .037* |
| MI, 2MWTwith Asymmetry | R^2^ = .058; F(1.36) = 2.214; p = .145 | Adj. R^2^ = .011; F(2.35) = 1.204; p = .312 |  |
| MI, 2MWTwith Postural stability | R^2^ = .058; F(1.36) = 2.214; p = .145 | Adj. R^2^ = .006; F(2.35) = 1.117; p = .339 |  |
| MI, 2MWTwithout Tempo | R^2^ = .026; F(1.32) = .856; p = .362 | Adj. R^2^ = .028; F(2.31) = 1.475; p = .244 |  |
| MI, 2MWTwithout Symmetry | R^2^ = .024; F(1.31) = .757; p = .391 | Adj. R^2^ = .157; F(2.30) = 3.983; p = .029* | ∆R^2^ = .186; ∆F(1.30) = 7.061; p = .013* |
| MI, 2MWTwithout Postural stability | R^2^ = .026; F(1.32) = .856; p = .362 | Adj. R^2^ = -.037; F(2.31) = .417; p = .662 |  |
| TCT, SIT Path | R^2^ = .416; F(1.51) = 36.261; p < .001* | Adj. R^2^ = .420; F(2.50) = 19.859; p < .001* | ∆R^2^ = .027; ∆F(1.50) = 2.436; p = .125 |
| TCT, SIT FOAM Path | R^2^ = .291; F(1.44) = 18.092; p < .001* | Adj. R^2^ = .318; F(2.43) = 11.486; p < .001* | ∆R^2^ = .057; ∆F(1.43) = 3.749; p = .059 |
| TCT, STAND Path | R^2^ = .144; F(1.37) = 6.220; p = .017* | Adj. R^2^ = .129; F(2.36) = 3.808; p = .032* | ∆R^2^ = .031; ∆F(1.36) = 1.340; p = .255 |
| TCT, STAND EC Path | R^2^ = .088; F(1.29) = 2.789; p = .106 | Adj. R^2^ = .045; F(2.28) = 1.702; p = .201 |  |
| TCT, STAND FOAM Path | R^2^ = .083; F(1.21) = 1.894; p = .183 | Adj. R^2^ = .162; F(2.20) = 3.121; p = .066 |  |
| TCT, 2MWTwith Tempo | R^2^ = .058; F(1.39) = 2.392; p = .130 | Adj. R^2^ = .154; F(2.38) = 4.653; p = .016* | ∆R^2^ = .139; ∆F(1.38) = 6.573; p = .014* |
| TCT, 2MWTwith Asymmetry | R^2^ = .058; F(1.39) = 2.392; p = .130 | Adj. R^2^ = .008; F(2.38) = 1.165; p = .323 |  |
| TCT, 2MWTwith Postural stability | R^2^ = .058; F(1.39) = 2.392; p = .130 | Adj. R^2^ = .020; F(2.38) = 1.408; p = .257 |  |
| TCT, 2MWTwithout Tempo | R^2^ = .170; F(1.34) = 6.943; p = .013* | Adj. R^2^ = .128; F(2.33) = 3.576; p = .039* | ∆R^2^ = .009; ∆F(1.33) = .344; p = .562 |
| TCT, 2MWTwithout Symmetry | R^2^ = .173; F(1.33) = 6.884; p = .013* | Adj. R^2^ = .214; F(2.32) = 5.620; p = .008* | ∆R^2^ = .087; ∆F(1.32) = 3.776; p = .061 |
| TCT, 2MWTwithout Postural stability | R^2^ = .170; F(1.34) = 6.943; p = .013* | Adj. R^2^ = .127; F(2.33) = 3.540; p = .040* | ∆R^2^ = .007; ∆F(1.33) = .283; p = .598 |
| BBS, SIT Path | R^2^ = .399; F(1.53) = 35.117; p < .001* | Adj. R^2^ = .382; F(2.52) = 17.692; p < .001* | ∆R^2^ = .006; ∆F(1.52) = .558; p = .458 |
| BBS, SIT FOAM Path | R^2^ = .237; F(1.46) = 14.255; p < .001* | Adj. R^2^ = .262; F(2.45) = 9.334; p < .001* | ∆R^2^ = .057; ∆F(1.45) = 3.606; p = .064 |
| BBS, STAND Path | R^2^ = .063; F(1.38) = 2.545; p = .119 | Adj. R^2^ = .062; F(2.37) = 2.295; p = .115 |  |
| BBS, STAND EC Path | R^2^ = .011; F(1.30) = .326; p = .572 | Adj. R^2^ = -.024; F(2.29) = .633; p = .538 |  |
| BBS, STAND FOAM Path | R^2^ = .296; F(1.21) = 8.816; p = .007* | Adj. R^2^ = .289; F(2.20) = 5.476; p = .013* | ∆R^2^ = .058; ∆F(1.20) = 1.801; p = .195 |
| BBS, 2MWTwith Tempo | R^2^ = .179; F(1.40) = 8.710; p = .005* | Adj. R^2^ = .181; F(2.39) = 5.538; p = .008* | ∆R^2^ = .042; ∆F(1.39) = 2.121; p = .153 |
| BBS, 2MWTwith Asymmetry | R^2^ = .179; F(1.40) = 8.710; p = .005* | Adj. R^2^ = .196; F(2.39) = 5.989; p = .005* | ∆R^2^ = .056; ∆F(1.39) = 2.862; p = .099 |
| BBS, 2MWTwith Postural stability | R^2^ = .179; F(1.40) = 8.710; p = .005* | Adj. R^2^ = .183; F(2.39) = 4.367; p = .019* | ∆R^2^ = .004; ∆F(1.39) = .199; p = .658 |
| BBS, 2MWTwithout Tempo | R^2^ = .034; F(1.33) = 1.177; p = .286 | Adj. R^2^ = .007; F(2.32) = 1.115; p = .340 |  |
| BBS, 2MWTwithout Symmetry | R^2^ = .040; F(1.32) = 1.323; p = .258 | Adj. R^2^ = .075; F(2.31) = 2.337; p = .113 |  |
| BBS, 2MWTwithout Postural stability | R^2^ = .034; F(1.33) = 1.177; p = .286 | Adj. R^2^ = -.024; F(2.32) = .594; p = .558 |  |
| ** = p-value ≤ .05; MI = Motricity Index; TCT = Trunk Control Test; BBS = Berg Balance Scale; see table 1 for explanation of each inertial measurement unit-based test* | | | |
