# Supplementary material for: The added value of sensor-based tests in explaining the variance in walking and ADL independency after stroke: An exploratory study
Source: Clin Rehabil. 2025 Aug 3;39(10):1366–77. doi: 10.1177/02692155251362742 (PMC12414103; doi:10.1177/02692155251362742)
Supplement: sj-docx-5-cre-10.1177_02692155251362742 - Supplemental material for The added value of sensor-based tests in explaining the variance in walking and ADL independency after stroke: An exploratory study [file sj-docx-5-cre-10.1177_02692155251362742.docx]

#### **Supplementary material 4.**

Table 8. Multivariable regression analyses to explore the added value of inertial measurement unit-measured tests besides the conventional measures MI, TCT and BBS in explaining the variance in walking independence (determined by the Functional Ambulation Categories)

|  | **Model statistics** |  |  |  |  |  |
| --- | --- | --- | --- | --- | --- | --- |
| **Independent variables** | **Model 1 (conventional test)** | **Model 2 (conventional + inertial measurement unit-measured variable)** | **Change statistiscs model 2** |  |  |  |
| MI, SIT Path | R^2^ = .475; F(1.51) = 46.165; p < .001* | Adj. R^2^ = .462; F(2.50) = 23.352; p < .001* | ∆R^2^ = .008; ∆F(1.50) = .758; p = .388 |  |  |  |
| MI, SIT FOAM Path | R^2^ = .375; F(1.44) = 26.423; p < .001* | Adj. R^2^ = .356; F(2.43) = 13.418; p < .001* | ∆R^2^ = .009; ∆F(1.43) = .634; p = .430 |  |  |  |
| MI, STAND Path | R^2^ = .325; F(1.38) = 18.336; p < .001* | Adj. R^2^ = .297; F(2.37) = 9.221; p = .001* | ∆R^2^ = .007; ∆F(1.37) = .397; p = .533 |  |  |  |
| MI, STAND EC Path | R^2^ = .274; F(1.30) = 11.323; p = .002* | Adj. R^2^ = .250; F(2.29) = 6.156; p = .006* | ∆R^2^ = .024; ∆F(1.29) = .992; p = .328 |  |  |  |
| MI, STAND FOAM Path | R^2^ = .276; F(1.21) = 7.993; p = .010* | Adj. R^2^ = .241; F(2.20) = 4.487; p = .025* | ∆R^2^ = .034; ∆F(1.20) = .986; p = .332 |  |  |  |
| MI, 2MWTwith Tempo | R^2^ = .071; F(1.39) = 2.960; p = .093 | Adj. R^2^ = .233; F(2.38) = 7.081; p = .002* | ∆R^2^ = .201; ∆F(1.38) = 10.482; p = .003* |  |  |  |
| MI, 2MWTwith Asymmetry | R^2^ = .071; F(1.39) = 2.960; p = .093 | Adj. R^2^ = .103; F(2.38) = 3.291; p = .048* | ∆R^2^ = .077; ∆F(1.38) = 3.438; p = .072 |  |  |  |
| MI, 2MWTwith Postural stability | R^2^ = .071; F(1.39) = 2.960; p = .093 | Adj. R^2^ = .022; F(2.38) = 1.460; p = .245 |  |  |  |  |
| MI, 2MWTwithout Tempo | R^2^ = .003; F(1.33) = .111; p = .741 | Adj. R^2^ = .123; F(2.32) = 3.388; p = .046* | ∆R^2^ = .171; ∆F(1.32) = 6.646; p = .015* |  |  |  |
| MI, 2MWTwithout Symmetry | R^2^ = .000; F(1.32) = .003; p = .955 | Adj. R^2^ = -.062; F(2.31) = .032; p = .968 |  |  |  |  |
| MI, 2MWTwithout Postural stability | R^2^ = .003; F(1.33) = .111; p = .741 | Adj. R^2^ = -.059; F(2.32) = .055; p = .947 |  |  |  |  |
| TCT, SIT Path | R^2^ = .403; F(1.54) = 36.504; p < .001* | Adj. R^2^ = .384; F(2.53) = 18.112; p < .001* | ∆R^2^ = .003; ∆F(1.53) = .237; p = .629 |  |  |  |
| TCT, SIT FOAM Path | R^2^ = .340; F(1.47) = 24.265; p < .001* | Adj. R^2^ = .322; F(2.46) = 12.420; p < .001* | ∆R^2^ = .010; ∆F(1.46) = .720; p = .401 |  |  |  |
| TCT, STAND Path | R^2^ = .217; F(1.40) = 11.095; p = .002* | Adj. R^2^ = .204; F(2.39) = 6.257; p = .004* | ∆R^2^ = .026; ∆F(1.29) = 1.328; p = .256 |  |  |  |
| TCT, STAND EC Path | R^2^ = .166; F(1.32) = 6.382; p = .017* | Adj. R^2^ = .132; F(2.31) = 3.501; p = .043* | ∆R^2^ = .018; ∆F(1.31) = .683; p = .415 |  |  |  |
| TCT, STAND FOAM Path | R^2^ = .165; F(1.23) = 4.531; p = .044* | Adj. R^2^ = .172; F(2.22) = 3.492; p = .048* | ∆R^2^ = .076; ∆F(1.22) = 2.214; p = .151 |  |  |  |
| TCT, 2MWTwith Tempo | R^2^ = .026; F(1.42) = 1.114; p = .297 | Adj. R^2^ = .170; F(2.41) = 5.401; p = .008* | ∆R^2^ = .183; ∆F(1.41) = 9.463; p = .004* |  |  |  |
| TCT, 2MWTwith Asymmetry | R^2^ = .026; F(1.42) = 1.114; p = .297 | Adj. R^2^ = .073; F(2.41) = 2.696; p = .079 |  |  |  |  |
| TCT, 2MWTwith Postural stability | R^2^ = .026; F(1.42) = 1.114; p = .297 | Adj. R^2^ = -.021; F(2.41) = .554; p = .579 |  |  |  |  |
| TCT, 2MWTwithout Tempo | R^2^ = .075; F(1.35) = 2.845; p = .101 | Adj. R^2^ = .109; F(2.34) = 3.213; p = .053 |  |  |  |  |
| TCT, 2MWTwithout Symmetry | R^2^ = .098; F(1.34) = 3.702; p = .063* | Adj. R^2^ = .067; F(2.33) = 2.256; p = .121 |  |  |  |  |
| TCT, 2MWTwithout Postural stability | R^2^ = .075; F(1.35) = 2.845; p = .101 | Adj. R^2^ = .042; F(2.34) = 1.791; p = .182 |  |  |  |  |
| BBS, SIT Path | R^2^ = .634; F(1.56) = 97.205; p < .001* | Adj. R^2^ = .635; F(2.55) = 50.629; p < .001* | ∆R^2^ = .014; ∆F(1.55) = 2.116; p = .151 |  |  |  |
| BBS, SIT FOAM Path | R^2^ = .535; F(1.49) = 56.314; p < .001* | Adj. R^2^ = .517; F(2.48) = 27.811; p < .001* | ∆R^2^ = .002; ∆F(1.48) = .213; p = .646 |  |  |  |
| BBS, STAND Path | R^2^ = .344; F(1.41) = 21.487; p < .001* | Adj. R^2^ = .334; F(2.40) = 11.546; p < .001* | ∆R^2^ = .022; ∆F(1.40) = 1.397; p = .244 |  |  |  |
| BBS, STAND EC Path | R^2^ = .285; F(1.33) = 13.162; p = .001* | Adj. R^2^ = .245; F(2.32) = 6.528; p = .004* | ∆R^2^ = .005; ∆F(1.32) = .210; p = .650 |  |  |  |
| BBS, STAND FOAM Path | R^2^ = .412; F(1.23) = 16.127; p < .001* | Adj. R^2^ = .369; F(2.22) = 8.014; p = .002* | ∆R^2^ = .009; ∆F(1.22) = .355; p = .558 |  |  |  |
| BBS, 2MWTwith Tempo | R^2^ = .440; F(1.43) = 33.743; p < .001* | Adj. R^2^ = .427; F(2.42) = 17.417; p < .001* | ∆R^2^ = .014; ∆F(1.42) = 1.050; p = .311 |  |  |  |
| BBS, 2MWTwith Asymmetry | R^2^ = .440; F(1.43) = 33.743; p < .001* | Adj. R^2^ = .414; F(2.42) = 16.573; p < .001* | ∆R^2^ = .001; ∆F(1.42) = .106; p = .747 |  |  |  |
| BBS, 2MWTwith Postural stability | R^2^ = .440; F(1.43) = 33.743; p < .001* | Adj. R^2^ = .415; F(2.42) = 16.582; p < .001* | ∆R^2^ = .002; ∆F(1.42) = .115; p = .736 |  |  |  |
| BBS, 2MWTwithout Tempo | R^2^ = .080; F(1.34) = 2.966; p = .094 | Adj. R^2^ = .112; F(2.33) = 3.205; p = .053 |  |  |  |  |
| BBS, 2MWTwithout Symmetry | R^2^ = .143; F(1.33) = 5.507; p = .025* | Adj. R^2^ = .112; F(2.32) = 3.140; p = .057 |  |  |  |  |
| BBS, 2MWTwithout Postural stability | R^2^ = .080; F(1.34) = 2.966; p = .094 | Adj. R^2^ = .027; F(2.33) = 1.479; p = .243 |  |  |  |  |
| ** = p-value ≤ .05; MI = Motricity Index; TCT = Trunk Control Test; BBS = Berg Balance Scale; see table 1 for explanation of each inertial measurement unit-based test* | | | | |  |  |
